# Positron emission tomography dataset of [^11^C]carbon dioxide storage in coal for geo-sequestration application

**DOI:** 10.1038/s41597-023-02754-3

**Published:** 2023-11-29

**Authors:** Yu Jing, Aaron Uthaia Kumaran, Damion Howard Read Stimson, Karine Mardon, Ljubco Najdovski, Ryan T. Armstrong, Peyman Mostaghimi

**Affiliations:** 1 School of Minerals and Energy Resources Engineering, The University of New South Wales, Sydney, NSW 2052 Australia; 2 Centre for Advanced Imaging, Australian Institute for Bioengineering and Nanotechnology, University of Queensland, Brisbane, QLD 4072 Australia

**Keywords:** Geodynamics, Geophysics, Petrology

## Abstract

Positron Emission Tomography (PET) imaging has demonstrated its capability in providing time-lapse fluid flow visualisation for improving the understanding of flow properties of geologic media. To investigate the process of CO_2_ geo-sequestration using PET imaging technology, [^11^C]CO_2_ is the most optimal and direct radiotracer. However, it has not been extensively used due to the short half-life of Carbon-11 (20.4 minutes). In this work, a novel laboratory protocol is developed to use [^11^C]CO_2_ as radiolabelled tracer to visualise and quantify *in-situ* CO_2_ adsorption, spreading, diffusion, and advection flow in coal. This protocol consists of generation and delivering of [^11^C]CO_2_, lab-based PET scanning, subsequent micro-CT scanning, and data processing. The lab-based PET scanning setup integrates *in-situ* core flooding tests with PET scanning. The real-time PET images are acquired under different storage conditions, including early gas production stage, depleted stage, and late storage stage. These datasets can be used to study across-scale theoretical and experimental study of CO_2_ flow behaviour in coal with the application to CO_2_ geo-sequestration.

## Background & Summary

To achieve the net zero emission goal to mitigate the global warming issues, negative emission technologies must be deployed to remove carbon dioxide (CO_2_) from air or point sources and store it. CO_2_ geo-sequestration is one of the critical components in decarbonisation technologies for long-term and large-scale CO_2_ storage^1^. Feasible CO_2_ geological storage formations include depleted hydrocarbon reservoirs, saline aquifers, and coal seams. Compared with the former two options, CO_2_ storage in coal seams has a unique adsorption trapping mechanism, as CO_2_ can be permanently retained within coal seams in adsorbed state if the *in-situ* pressure is maintained. Therefore, CO_2_ geo-sequestration in coal seams could be safer with high storage capacity^2^. Due to the complexity and heterogeneity of coal, CO_2_ storage in coal seams is more complicated, which includes gas spreading, trapping, migrating, and interacting with coal matrix. These coupled physical phenomena are both spatially localised and time-dependant, which are yet thoroughly known. Thus, it is of importance to provide direct insights about the subsurface processes during CO_2_ geo-sequestration.

Micro-Positron Emission Tomography (micro-PET) can create four-dimensional images of the radiotracer transport, which has been used in visualising real-time fluid flow in rock cores due to its high temporal (≈10 seconds) resolution^3–6^. [^18^F]FDG (Fluorodeoxyglucose) is the most used aqueous radiotracer for solute transport study in porous media because of its availability and suitable radioisotope half-life (109 mins). However, for the application of CO_2_ geo-sequestration, we need to directly visualise gaseous CO_2_ flow dynamics in coal by using [^11^C]CO_2_ as the radiotracer. But radioisotope ^11^C is rarely used for the study of fluid flow in porous media, as it is difficult to complete radiotracer delivery and injection within its short active lifetime (half-life = 20.4 mins). The gaseous phase of [^11^C]CO_2_ further makes the radiotracer handling a challenge, only two studies are found from the literature^7,8^.

**Fig. 1:**
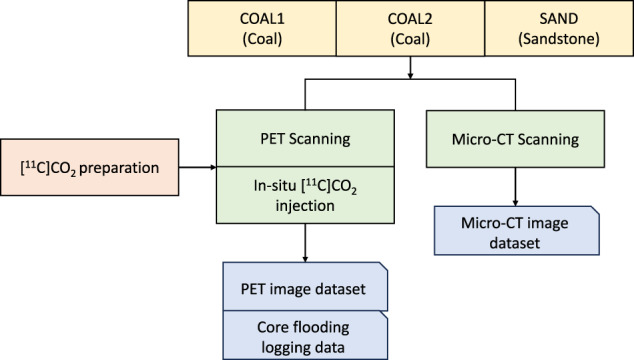
Schematic overview of the workflow. This experimental protocol integrates PET scanning with *in-situ* core flooding, to visualise CO_2_ storage process. [^11^C]CO_2_ is prepared at an on-site cyclotron and injected into three types of rocks as the radiotracer. PET image datasets and pressure data during core flooding tests are deposited publicly. In addition, micro-CT imaging technology is also applied for all three samples to obtain microscale pore/fracture features.

Herein, for the first time, we use ^11^C to trace CO_2_ gas flow in coal using PET scanning, aiming to measure temporal and spatial evolution of CO_2_ storage process in coal. The real-time PET images are acquired under different *in-situ* storage conditions, including early gas production stage, reservoir depleted stage, and late storage stage. In this data descriptor, the laboratory-based PET image acquisition method is described, including the radiotracer generation, delivery and handling, apparatus, and experimental design. All PET images and high-resolution micro-CT images of tested samples are publicly available on Figshare. Figure 1 shows a schematic overview of the workflow. This experimental protocol gives direct observation of CO_2_ gas adsorption, spreading, diffusion, and advection flow in coal. These datasets could be reused by other researchers to validate modelling methods and to study *in-situ* CO_2_ storage physics in coal for the application of CO_2_ geo-sequestration.

## Methods

### [^11^C]CO_2_ generation, entrapment, and delivery

**Fig. 2:**
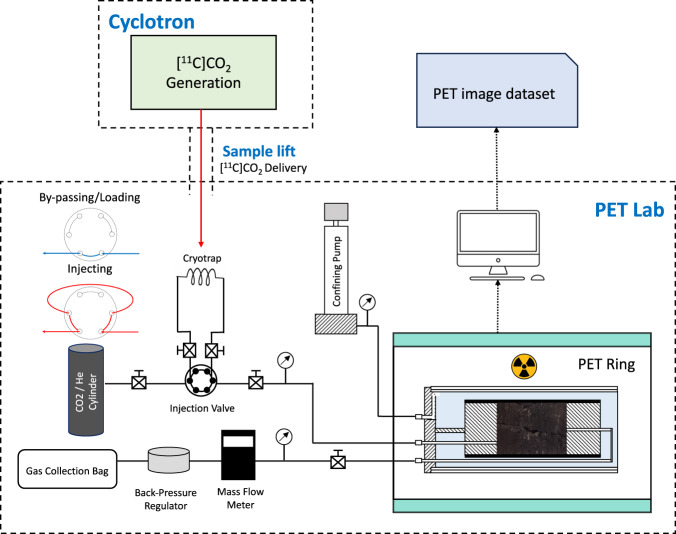
Schematic of the experimental setup. The cryotrap coil collects [^11^C]CO_2_ from the cyclotron. It is then transferred via a sample lift to the PET lab and is connected to the injection valve for loading (loading position). After loading, switch the valve to injecting position to start injecting [^11^C]CO_2_ into the core. The whole flowline is shielded.

Carbon-11 (^11^C), a radioisotope of carbon, has a half-life of 20.4 minutes. In this work, ^11^C is prepared as [^11^C]CO_2_ using an on-site cyclotron (“Cyclone-18” fixed energy cyclotron, Ion Beam Applications, Belgium) at the Centre for Advanced Imaging, University of Queensland. Generated [^11^C]CO_2_ is automatically delivered at 500 mL/min via a mass flow controller (Synthra, GmbH) and trapped in a cryotrap at - 195 °C within a shielded “hot-cell” (Tema Sinergie, Italy). The cryotrap is made of a coil of 1/8” OD stainless steel tubing with two ball valves at each end. Its internal volume is 3.64 mm^3^. A dose calibrator is installed within the hot-cell to measure the quantity of ^11^C in the coil. After trapping [^11^C]CO_2_ from the cyclotron, the two ball values of the cryotrap are closed to secure the trapped [^11^C]CO_2_. Next, the cryotrap coil, disconnected from the hot cell, is inserted into a shielded dewar containing liquid nitrogen, which is then delivered to the PET scanning lab via a specific sample lift (Fig. 2).

### Laboratory-based PET imaging setup

The experimental design integrates PET scanning with *in-situ* core flooding setup which enables imaging and gas injecting at the same time (Fig. 2). The scanning instrument is Siemens Inveon micro-PET/CT Platform, where the ring has 120 cm internal diameter and is 12 cm long. The core holder is specifically designed for the Siemens Inveon PET scanner, such that it can be positioned inside the PET scanner ring. The upstream flowline connects the sample inlet with gas sources (CO_2_, He, CH_4_ gas cylinders). Pressurised gas from the gas cylinders is pumped into the sample, measured at the outlet using a gas mass flowmeter, then collected at the end of the flowline in a shielded “Tedlar” gas capture bag (Grace Discovery Sciences, USA). A back-pressure regulator at the downstream is used to maintain a desired outlet pressure.

### [^11^C]CO_2_ loading

A dual-position six-port injection valve is installed at the upstream to load [^11^C]CO_2_ into the injection flowline. Initially, the gas is injected into the core directly when the injection valve is under loading position. Meanwhile, the cryotrap coil that collects [^11^C]CO_2_ from the hot-cell is connected to the injection valve. For loading the radiotracer, the injection valve is switched to the loading position, such that gas from the cylinder will flow through the cryotrap coil to mix [^11^C]CO_2_ into the injection stream.

### Samples and pre-treatments

Two coal samples and one Bentheimer sandstone core are studied in this work (D = 5.2 cm, L = 6 cm). One coal sample is relatively tight with fewer visible fractures, named “COAL1”. The other coal sample is banded, where the bright band is highly fractured and percolates along the length, named “COAL2”. Coal samples are pre-treated prior to CO_2_ injection to mimic different underground conditions, including dry condition, dewatered condition, and gas-saturated condition. The different initial conditions of the tests could represent various storage stages. For instance, dewatered coal sample with residual CH_4_ in place represents early-stage coal seam gas production condition, dewatered coal sample without residual CH_4_ represents depleted reservoir condition, CO_2_-saturated coal sample represents the late-stage of CO_2_ storage approaching storage capacity. The dewatered condition is achieved by injecting helium into a water-saturated sample to drain the water phase until no water is produced from the outlet to reach the residual water saturation. To saturate the sample, CO_2_ (or CH_4_) is pumped into the dry sample under desired pressures for at least 12 hours. For the dewatered condition with residual CH_4_ in place, we pump water into the CH_4_-saturated coal sample under the same gas pressure to keep the pore pressure constant, followed by releasing the outlet pressure to start dewatering and gas desorption.

### Experiment procedures

After the sample is pre-treated, carrier gas is injected under a constant gas pressure into the core under room temperature. When the pressures and outlet gas flowrate become stable, we load and inject [^11^C]CO_2_ and start PET scanning. In this work, there are 5 sets of core flooding tests, and 10 [^11^C]CO_2_ injections and corresponding PET scans. Table 1 lists details of each test, where gas flow rates, inlet and outlet pressures, confining pressure, and carrier gas are recorded.

**Table 1:** Overview of the core flooding experiments conducted under different conditions.

Test	Scan	Sample	Initial Condition	Carrier Gas	*P*_*con*_ [psi]	*P*_*in*_ [psi]	*P*_*out*_ [psi]	*Q*_*out*_ [L/min]	Scanning Time [min]
1	1–3	COAL1	Dewatered	CO_2_	225	48.9	41.0	0.02	30
2	4–5	COAL2	Dry	He	231	45.1	44.1	0.06	30
3	6–7	COAL2	Dewatered, CH_4_ saturated	CO_2_	205	81.9	39.5	0.36	30
4	8–9	COAL2	CO_2_ saturated	CO_2_	203	62.9	25.8	0.17	30
5	10	SAND	Dry	He	237	45.1	44.1	3.53	15

### PET scanning and image construction

PET scanning is performed simultaneously while injecting [^11^C]CO_2_. The scanning instrument is a Siemens Inveon preclinical PET/CT scanner at The Centre for Advanced Imaging, University of Queensland. The effective spatial resolution for the scanner is calculated to be 1.6 mm, which is quantitatively assessed during the scanner calibration but using a Fludeoxyglucose (^18^F) calibration source rather than a carbon-11 calibration source. The scanning data are reconstructed to create time lapse images, using OSEM2D (ordered subset expectation maximization 2D) method. Time frames can be less than 10 seconds, depending on the intensity of the radiotracer and scanner properties. In PET acquisition settings of this work, PET data are reconstructed at timeframes according to the gas breakthrough time, ranging from 2 s to 300 s. Table 2 lists the initial radio activity measured at the hot-cell and at the time of PET scanning.

**Table 2:** Initial radio activity and time framing of each PET scan.

Scan	Sample	Activity @ Hot-cell [MBq]	Activity @ Injection [MBq]
1	COAL1	745	78.93
2	COAL1	500	79.68
3	COAL1	450	97.39
4	COAL2	182	77.81
5	COAL2	117	67.89
6	COAL2	129	53.11
7	COAL2	189	121.84
8	COAL2	245	142.17
9	COAL2	221	128.25
10	SAND	222	58.92

## Data Records

All 10 sets of PET images have been deposited on Figshare^9^. To provide microscale pore/fracture features of samples, we also upload the high-resolution micro-CT images of two coal samples^10,11^, where the images are obtained by HeliScan micro-CT scanner (Mark 1, ThermoFisher) at the University of New South Wales. The micro-CT image datasets can be deployed to obtain quantitative information of porosity and permeability, which could be further used in tracer transport modelling to fit with measured PET image data. Apart from the image dataset, the logged pressure data of core flooding tests are also shared on Figshare^12^, including inlet, outlet, and confining pressures. Detailed information of each scan is provided in following tables.

## Technical Validation

**Fig. 3:**
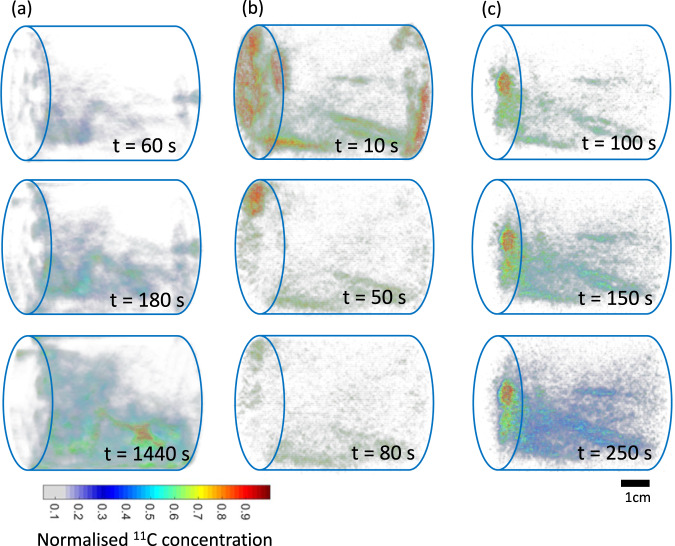
Selected PET images of three tests. Column (**a**) is from unsaturated and tight coal sample, COAL1; (**b**) is for saturated and fractured coal sample, COAL2; (**c**) is for unsaturated and fracture coal sample, COAL2. Time-lapsed PET images present CO_2_ spreading, diffusing, adsorbing, flowing, and breaking through the core.

All PET scans can visualise the CO_2_ spreading, diffusing, adsorbing, flowing, and breaking through the core. Some selected PET images are shown in Fig. 3. Column (a - c) are time-lapse PET images of three different tests. The breakthrough time, CO_2_ concentration and distribution differ between different tests, which is due to different initial sample conditions and sample types. Figure 3a shows results of the unsaturated and tight coal sample. In this case, CO_2_ storage is a slower process, where CO_2_ diffuses within the sample with a late gas breakthrough. While for unsaturated fractured coal sample (Fig. 3c), CO_2_ breakthrough happens first, followed by gas adsorption and diffusion. On the other hand, if the fractured coal sample has been saturated initially, CO_2_ gas quickly occupies fracture space and breaks through at the outlet (Fig. 3b). In our preliminary analysis, we estimate the dispersion coefficients for selected scans by fitting the experimental PET data with modelling data derived from Advection-Dispersion Equation (ADE). Scan 8 and 9 are repeat scanning for CO_2_-saturated coal sample under the same conditions with 2 hours apart. They give similar estimated dispersion coefficients at the magnitude of 10^−9^ cm^2^/min. More analysis and discussions could be found in our previous paper^13^, which could further validate the reliability of obtained image data.

## Usage Notes

All PET images have been cropped to the size of the true sample and converted to.tif format, which can be opened and analysed by most image analysing and visualisation tools, including open-source software ImageJ (Fiji), Thermo-Scientific Avizo, and ORS Dragonfly. Each image frame represents a specific time span, ranging from 2 s to 300 s (Table 3). Thus, the tracer activity should be averaged over the corresponding time frame:

$${A}_{j}^{* }\left({t}_{i}\right)=\frac{1}{\Delta t}{\int }_{{t}_{1}}^{{t}_{2}}{a}_{j}^{PET}\left(t\right)dt$$

where ${A}_{j}^{* }\left({t}_{i}\right)$ is the average tracer activity in each voxel *j* over a time frame Δ*t* = *t*_2_ − *t*_1_, *t*_i_ = (*t*_1_ + *t*_2_)/2. PET signal decay during the overall acquisition time is automatically corrected by the Inveon IAW software. But at the beginning of each scan, [^11^C]CO_2_ radioactivity varies since each time when it is made from the cyclotron has different radioactivity. In this work, we run 5 core-flooding tests and 10 scans (Table 1). When comparing different scans for the same test, it is necessary to know the exact radioactivity dose for each scan. The tracer activity measured at the hot-cell is known (Table 2), then the radioactivity when the scan is started can be calculated by accounting for radioactivity decay, ${A}_{{t}_{0}}^{inlet}={e}^{\lambda t}{A}_{{t}_{hot-cell}}$, where $\lambda =ln\left(2\right)/{t}_{1/2}$, *t*_1/2_ is half-life of ^11^C (20.4 mins), *t* is time since the radioactivity is measured at the hot-cell. Please refer to our previous paper^13^ for more details about data processing. The micro-CT images can be segmented and registered with corresponding PET images using software such as Thermo-Scientific Avizo, to obtain porosity and permeability map of each sample Table 4.

**Table 3:** Description of data records of PET image datasets, including file name, image size, voxel size, and frame timing.

Run	PET Image Dataset Details	Frame	Frame Length [s]
1	File name: PET_run1_coal1_dewatered_1	1–30	10
	Image size: 128 × 128 × 159	31–35	60
	Voxel size: 0.776 × 0.776 × 0.796 mm	36–39	300
2	File name: PET_run2_coal1_dewatered_2	1–30	10
	Image size: 128 × 128 × 159	31–35	60
	Voxel size: 0.776 × 0.776 × 0.796 mm	36–39	300
3	File name: PET_run3_coal1_dewatered_3	1–30	10
	Image size: 128 × 128 × 159	31–35	60
	Voxel size: 0.776 × 0.776 × 0.796 mm	36–39	300
4	File name: PET_run4_coal2_dry_1	1–30	10
	Image size: 128 × 128 × 159	31–35	60
	Voxel size: 0.776 × 0.776 × 0.796 mm	36–39	300
5	File name: PET_run5_coal2_dry_2	1–60	2
	Image size: 128 × 128 × 159	61–63	60
	Voxel size: 0.776 × 0.776 × 0.796 mm	64–68	300
6	File name: PET_run6_coal2_CH4_1	1–60	2
	Image size: 128 × 128 × 159	61–63	60
	Voxel size: 0.776 × 0.776 × 0.796 mm	64–68	300
7	File name: PET_run7_coal2_CH4_2	1–30	10
	Image size: 128 × 128 × 159	31–35	60
	Voxel size: 0.776 × 0.776 × 0.796 mm	36–39	300
8		1–3	300
	File name: PET_run8_coal2_CO2_12hr	4–7	60
	Image size: 128 × 128 × 159	8–43	5
	Voxel size: 0.776 × 0.776 × 0.796 mm	44–73	10
		74–79	30
9	File name: PET_run8_coal2_CO2_14hr	1–24	5
	Image size: 128 × 128 × 159	25–27	60
	Voxel size: 0.776 × 0.776 × 0.796 mm	28–32	300
10	File name: PET_run10_sand_dry	1–4	30
	Image size: 128 × 128 × 159	5–40	5
	Voxel size: 0.776 × 0.776 × 0.796 mm	71–75	60
		41–70	10

**Table 4:** Description of data records of micro-CT image datasets, including file name, image size, and resolution.

File Name	Sample	Image Size	Resolution
Micro-CT images of a coal sample (COAL1)	COAL1	2520 × 2520 × 2560	33 µm
Micro-CT images of a coal sample (COAL2)	COAL2	2520 × 2520 × 3162	26 µm

## Data Availability

This work does not require any code.
